# Understanding cell culture dynamics: a tool for defining protocol parameters for improved processes and efficient manufacturing using human embryonic stem cells

**DOI:** 10.1080/21655979.2021.1902696

**Published:** 2021-03-24

**Authors:** J.W.T. Kusena, M. Shariatzadeh, R.J. Thomas, S.L. Wilson

**Affiliations:** Centre for Biological Engineering, Wolfson School of Mechanical, Electrical and Manufacturing Engineering, Loughborough University, Epinal Way, Loughborough University, Loughborough, Leicestershire, UK

**Keywords:** Cell therapy, process development, protocol standardization, regenerative medicine

## Abstract

Standardization is crucial when culturing cells including human embryonic stem cells (hESCs) which are valuable for therapy development and disease modeling. Inherent issues regarding reproducibility of protocols are problematic as they hinder translation to good manufacturing practice (GMP), thus reducing clinical efficacy and uptake. Pluripotent cultures require standardization to ensure that input material is consistent prior to differentiation, as inconsistency of input cells creates end-product variation. To improve protocols, developers first must understand the cells they are working with and their related culture dynamics. This innovative work highlights key conditions required for optimized and cost-effective bioprocesses compared to generic protocols typically implemented. This entailed investigating conditions affecting growth, metabolism, and phenotype dynamics to ensure cell quality is appropriate for use. Results revealed critical process parameters (CPPs) including feeding regime and seeding density impact critical quality attributes (CQAs) including specific metabolic rate (SMR) and specific growth rate (SGR). This implied that process understanding, and control is essential to maintain key cell characteristics, reduce process variation and retain CQAs. Examination of cell dynamics and CPPs permitted the formation of a defined protocol for culturing H9 hESCs. The authors recommend that H9 seeding densities of 20,000 cells/cm^2^, four-day cultures or three-day cultures following a recovery passage from cryopreservation and 100% medium exchange after 48 hours are optimal. These parameters gave ~SGR of 0.018 hour^−1^ ± 1.5x10^−3^ over three days and cell viabilities ≥95%±0.4, while producing cells which highly expressed pluripotent and proliferation markers, Oct3/4 (>99% positive) and Ki-67 (>99% positive).

## Graphical abstract

**Figure:**
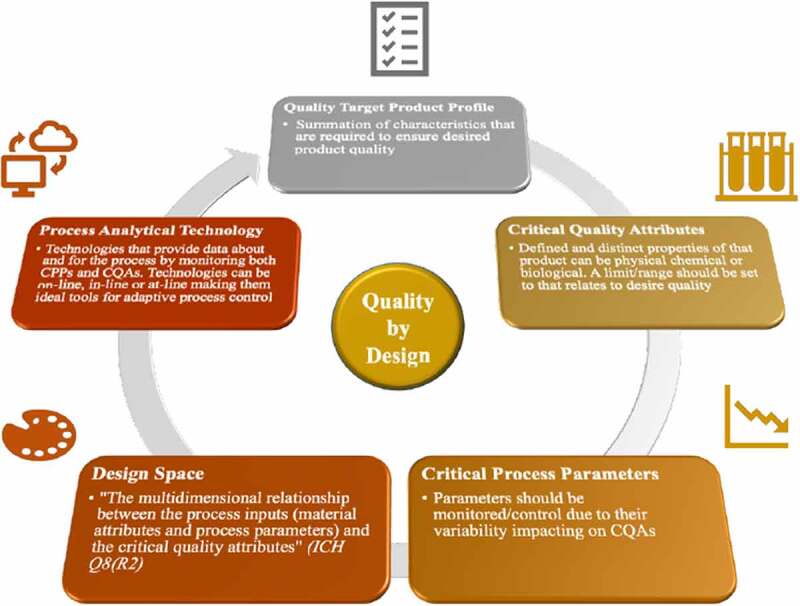

## Introduction

Following decades of research, there are a plethora of cell-based therapies traversing the different phases of clinical trials [1–7]. However, prior to clinical realization, there is a requirement for well-defined protocols to ensure comparability and reproducibility, since in many cases effective comparisons of data can be encumbered by the lack of standardized culture protocols [8–10]. These protocols have a propensity to overlook critical process parameters (CCPs) such as medium exchange volume, feeding regime frequency and cell seeding density. This lack of standardization often results in variation of the measured process outputs such as the critical quality attributes (CQAs) of the product including growth rates, metabolism, and cell phenotype. This results in challenges for obtaining regulatory approval as regulators demand that products are well characterized, and processes are robust, controlled and well understood [11–14]. Achieving this requires developmental work to be performed, to understand the complex cell dynamics and to standardize protocols for the manufacture of cell therapy products (CTPs) [15]. These protocols must provide manufacturing processes that can withstand the high scrutiny of regulatory agencies including the Food and Drug Administration (FDA) and European Medicines Agency (EMA) [16,17]. Several studies have reported that the variation between independent hESC lines is due to differences in culture protocols [18] including feeder cell types and seeding densities, culture substrates, culture media, growth factors/other media additives and passage methods used [19]. These studies however focused solely on the implementation of serum-free culture media, growth factors and other molecular modulators as opposed to key culture parameters and variables that can affect the cell growth dynamics and quality and dictate H9 suitability for their clinical applications.

This is pertinent as pluripotent human embryonic stem cells (hESCs) have been used as input material for a plethora of disease models and CTPs including Alzheimer’s Disease, Parkinson’s Disease, diabetes and cardiac diseases [20–25]. Currently, H9 hESCs are being used as input cell material for differentiation into ventral mesencephalic dopaminergic (vmDA) neuroprogenitors by groups internationally, for Parkinson’s disease (PD) CTP development and research [1,26–28]. H9 hESCs were used in the present work to illustrate the effects of cell seeding density and feeding regimes on characteristics such as cell viability, specific growth rate (SGR) and phenotype. The rationale for this study was to highlight the importance of understanding cell culture dynamics which are imperative to process understanding. This is crucial for clinically relevant cell lines including H9s, as they should be well defined prior to their use as the input material for a CTP. This is necessary for the facilitation of more efficient manufacturing processes that produce the desired cell numbers and characteristics with less variation and, ultimately providing products that are better placed for both regulatory approval and reimbursed adoption.

The specific objectives and aim of this work were to understand the culture dynamics of pluripotent H9 hESCs. This is significant since the understanding of key culture parameters and the variables that can affect the quality of the H9 cell line which are currently massively under-presented/scarce, the novel knowledge gained from these findings will be employed to provide better defined and improved protocol parameters for hESC bioprocessing. These optimized and streamlined protocols comprising well-defined feeding regimes and initial seeding density can subsequently be applied in expansion of H9 cell lines prior to dopaminergic neuroprogenitor differentiation, whilst providing greater resource, time, and cost efficiency.

It was hypothesized that by gaining a detailed understanding of the cells’ growth behavior and characteristics from the onset, protocols can be established that adequately control for efficient and reproducible growth of input cell material. The input cell material can then be used for further cell processing steps such as differentiation to specific lineages, e.g., neural or hematopoietic lineages [29,30]. The purpose is to develop protocols that provide measurable metrics for operators to use as benchmarks of quality; these could include minimum cell viability levels, SGR, and expression of phenotypic markers, that are needed to qualify a pluripotent expansion culture as a source of ‘good-quality’ starting material, for instance. This is important since an improved understanding of the cell growth dynamics may result in more efficient and defined protocols with optimized feeding regimes. Consequently, a reduction in the use of resources including reagents, labor and operator/laboratory/equipment time, are desirable from a manufacturing process perspective since it reduces costs and output variation due to reduced operator interventions.

## Materials and methods

The laboratory setting where all experiments were undertaken run under an industry-style system whereby different cell cultures are segregated into defined areas and equipment with robust cleaning and maintenance protocols in place. The facility operates under a quality-based system based on ISO 9001:2015 quality system principles [31].

## In *Vitro* cell culture

All reagents/consumables were obtained from Miltenyi Biotec (Surrey, UK), unless otherwise stated. Clinically relevant H9 hESCs (WiCell, cat# hPSCreg WAe009-A) were obtained from WiCell Research Institute, Incorporated (Madison, USA). H9s were thawed from cryopreservation and cultured on Biolaminin-521 (cat#LN521, BioLamina, Sweden) coated tissue culture plastic-ware (TCP) at a concentration of 0.5 μg/cm^2^ at 37°C, 5% CO_2_, using StemMACS™ iPS-Brew XF (cat#130-104-368) supplemented with 2.0% *vol/vol* of StemMACS™ iPS-Brew XF supplement (50X), herein referred to as growth medium. Rho kinase inhibitor (ROCK*i*, Y-27,632 10 μM) was used to supplement the growth medium during cell seeding and passaging. Cells were dissociated using Ethylenediaminetetraacetic acid (EDTA, cat#15,575,020, Thermo Fisher Scientific, Loughborough, UK) 75 μL/cm^2^ for 7 min at 37°C, 5% CO_2_.

### Experiment A: the effect of cell seeding density

**Figure 1.**
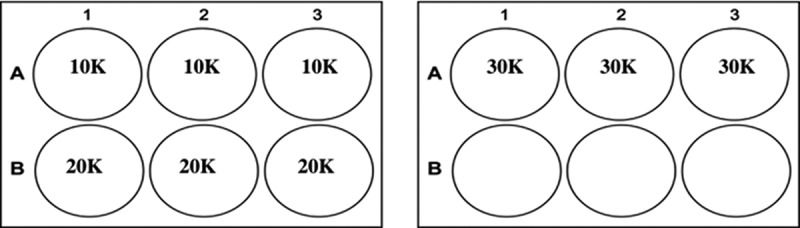
Experimental setup used to determine the growth curve of three different seeding densities: 10,000 cells/cm2 (10 K); 20,000 cells/cm2 (20 K) and 30,000 cells/cm2 (30 K). *n = *3 for each condition

H9 cells were thawed and seeded directly from cryopreservation at three densities (10,000; 20,000 and 30,000 cells/cm^2^) in triplicate onto Biolaminin-521 coated 6-well TCP (Figure 1). The cells were cultured for four days prior to passage and subjected to 100% growth medium exchange every 24 h (250 μL/cm^2^). The cells were analyzed at day two and day four for viability and SGR. Cell phenotype was analyzed at the end of the culture period. A four-day culture period was utilized as prior in-house experiments had demonstrated this to be an optimal period for the cells to reach confluency as observed by the plateau in SGR and cross validation with visual inspection of confluency.

### Experiment B: the effect of feeding regimes

To investigate the effect of different feeding regimes, the cells were seeded into four different routes of culture as detailed in Table 1 and cultured for two passages in triplicate for each route. Prior to the start of the experiment, the cells were cultured for one passage at 20,000 cells/cm^2^ in a Biolaminin-521 coated T25 cm^2^ flask. The cells were then harvested and reseeded into the four different routes of culture at 20,000 cells/cm^2^ onto Biolaminin-521 coated 6-well TCP. The cells were analyzed for viability, SGR, SMR and phenotype at the end of each passage.

**Table 1.** Details of the different experimental culture routes investigated in experiment B: the effect of feeding regimes. The **Control** route was subjected to daily medium exchanges; **Route 1** was subjected to a medium exchange on day one only; **Route 2** underwent a medium exchange on day 2; **Route 3** cells had no medium exchange. n = 3 for each condition was used. M. Ex = medium exchange

Route	Day 0	Day 1	Day 2	Day 3	Summary
Control	Seed	M.Ex	M.Ex	Passage/Harvest	Daily M.Ex
1	Seed	M.Ex		Passage/Harvest	M.ExEx on day 1 only
2	Seed		M.Ex	Passage/Harvest	M.Ex on day 2 only
3	Seed			Passage/Harvest	No M.Ex

For the second run* of the experiment, sacrificial wells for each of the four routes were seeded directly from cryopreservation for daily harvests, the experiment was performed in triplicate for each route. The daily harvests afforded a higher resolution experiment compared to the previous experiment where the data was collected at the end of each passage, here the data was collected daily for both passages. The cells were analyzed daily for viability, SGR and SMR; cell phenotype was analyzed at the end of each passage.

* Here a ‘run’ refers to a repeat of the experiment using the same experimental conditions with a different stock of input cell material.

**N.B**. The cells were cultured for three days instead of four days due to them being highly confluent by day three following one passage post-thaw prior to the start of the experiment.

### Experiment C: the effect of medium exhaustion

To investigate the effect of density and nutrient availability on cell growth inhibition, two culture conditions: daily feed (DF) and one feed (OF) of culture were set up for a seven-day culture period (Table 2). The DF condition was subjected to medium exchange every 24 h, whilst the OF condition was only subjected to a single medium exchange 24 h following initial seeding. Cells were thawed and seeded at 20,000 cells/cm^2^ onto Biolaminin-521 coated 6-well tissue culture microtiter plates. Sacrificial wells for each condition were seeded for daily harvests, the experiments were performed in triplicate for both conditions. The cells were analyzed daily for viability, SGR and SMR; cell phenotype was analyzed on day 0 and 7.

**Table 2.** Details of the two conditions used to investigate density and nutrient availability-based growth inhibition. Cells cultured following the Daily Feed (DF) route underwent medium exchange every 24 h; cells cultured under the one feed (OF) route underwent a single medium exchange following the first 24 h culture Sacrificial wells were harvested and counted daily: *n*= 3 for each condition was used. M. Ex = medium exchange; DF = daily feed; OF = one feed

Route	Day 0	Day 1	Day 2	Day 3	Day 4	Day 5	Day 6	Day 7
Daily Feed	Seed	M.Ex	M.Ex	M.Ex	M.Ex	M.Ex	M.Ex	Endpoint harvest
One Feed	Seed	M.Ex						Endpoint harvest

**N.B**. the cells were cultured for seven days (more than double the standard culture length of three days) to push the cells to their growth and metabolic limits using the OF route.

Cell counting

Cell counts and viability (*via* acridine orange uptake and 4′,6-diamidino-2-phenylindole (DAPI) exclusion) were obtained using an automated mammalian cell counter (NucleoCounter NC-3000, Chemometec, Denmark). Samples were analyzed using Via1-Cassettes and NC-Slide A8 slides (Chemometec, Denmark) using Solution 13 according to the manufacturer’s instructions to stain the cells for a Viability and Cell Count Assay (Chemometec, Denmark). The results were used to obtain specific growth rate (SGR) and Population Doublings (P_d_) using equations 1 and 2 published by Heathman *et al*., 2015^32^.

## Metabolite analysis

Spent media samples, 500 µL, were collected prior to cells being subjected 100% medium exchange then stored at −20°C prior to analysis. Spent media was analyzed for lactate, glucose, and lactate dehydrogenase (LDH) using the Cedex Bio-HT (Roche, Germany). The results were used to obtain the Specific Metabolite Rate mmol.cell^−1^.d^−1^ (SMR) using an equation published by Heathman *et al*., 2015^32^.

## Flow cytometry

Cells from each of the experimental conditions were resuspended in 1 mL of fixation solution per 10_6_ cells and incubated for 30 min at 4°C, to preserve them in their biological state at the point of harvest. The cells were then washed with 1 mL of cold protein extraction buffer (PEB) and centrifuged for 5 min at 300xG. A second wash step was performed with 1 mL of permeabilization buffer. The cells were then resuspended in 110 μL of staining master mix and incubated for 30 min at 4°C to permeabilize and stain the cells. The master mix comprise 80 μL permeabilization buffer, 10 μL Oct3/4-APC, 10 μL PAX6-PE and 10 μL Ki67-FITC conjugated antibodies. The cells were then washed with 1 mL permeabilization buffer and centrifuged for 5 min at 300xG. The cells were then resuspended in PEB buffer and 250 μL of each sample was analyzed using flow cytometry (BD FACSCanto^TM^ II, BD Biosciences, USA). All steps of the process were performed away from light.

The marker Oct3/4 was chosen due to it being a putative pluripotency marker and similar for Ki-67 as a proliferation marker; thus, expression of these markers should be high for cells in the pluripotent state [32,33]. Pax6 was added to the panel as it is an early marker for neuroectoderm differentiation used in the vmDA neuroprogenitor protocol; thus, it should be negative expression of Pax6 for cells in the pluripotent state [34–36].

Statistical Analysis

Unless otherwise noted, statistical significance was determined by two-way analysis of variance using Graphpad Prism Version 7.0d (CA, USA). Statistical significance was assigned as indicated in the legends. ‘*’ indicates p < 0.05, ‘**’ indicates p < 0.01, ‘***’ indicates p < 0.001, and ‘****’ indicates p < 0.0001. Tukey and Sidak’s multiple comparisons tests were used to compare means between groups.

## Results

The aim of this work was to understand the culture dynamics of pluripotent cells, with H9 hESCs used as an exemplar to provide defined protocol parameters that can be used for the expansion phase prior to differentiation toward lineages such as ventral mesencephalic dopaminergic (vmDA) neuroprogenitors [1,26–28]. Here the authors used the Lund University (vmDA) neuroprogenitors [1,26–28] differentiation process, focusing on the expansion phase of the pluripotent cells.

## Experiment A: the influence of seeding density during pluripotent Cell expansion

**Figure 2.**
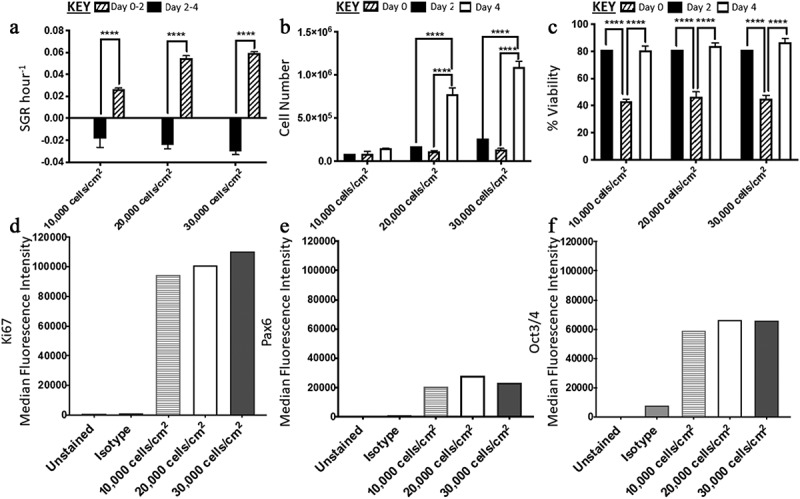
Analysis of H9 human embryonic stem cells (hESCs) growth dynamics and median fluorescence intensity (MedFI) values for OCT3/4, Ki67 and PAX6 markers for cells seeded at three different densities. (a) Specific growth rate (SGR) increased from negative SGR to positive SGR from day two to day four for all three densities. (b) Cell number decreased from day 0 to day two and then increased from day two four, the 10,000 cell/cm^2^ density did not have a significant increase in cell number during the four-day culture period. (c) All three densities had a decrease in cell viability from day 0 to day two which increased from day two four. OCT3/4 (d) and Ki67(f) were both highly expressed. (e) PAX6 expression levels were lower in comparison to OCT3/4 and Ki67. Error bars indicate standard deviation, *n = *3. ‘*’ indicates p < 0.05, ‘**’ indicates p < 0.01, ‘***’ indicates p < 0.001, and ‘****’ indicates p < 0.0001

There was no significant difference between the conditions in terms of their SGR from day 0 to 2. From day two to four, there were significant differences in SGR when comparing the 10,000 cells/cm^2^ condition to both the 20,000 and 30,000 cells/cm^2^ conditions, with the latter two being higher (Figure 2a). Furthermore, there was no significant difference in SGR overall between 20,000 and 30,000 cells/cm^2^ at the harvest point on day four. For each condition, there was a significant increase in SGR from day two to four. In terms of cell yield, only the 20,000 and 30,000 cells/cm^2^ conditions had significant increase from day 0 to day two and day two to four (Figure 2b). The cell viabilities across all conditions dropped significantly from day 0 to 2 from ~80% to ~40% and then increased from ~40% to ~80% by day four (Figure 2c). The median fluorescence intensity (MedFI) for Ki67 was the highest across the three markers analyzed (Figure 2d), there was expression of Pax6 observed in all three conditions (over 98% of cells were lowly expressing Pax6) (Figure 2e), and all conditions highly expressed Oct3/4 (87–97% positive) (Figure 2f),

## Experiment B: streamlining of feeding regimes

**Figure 3.**
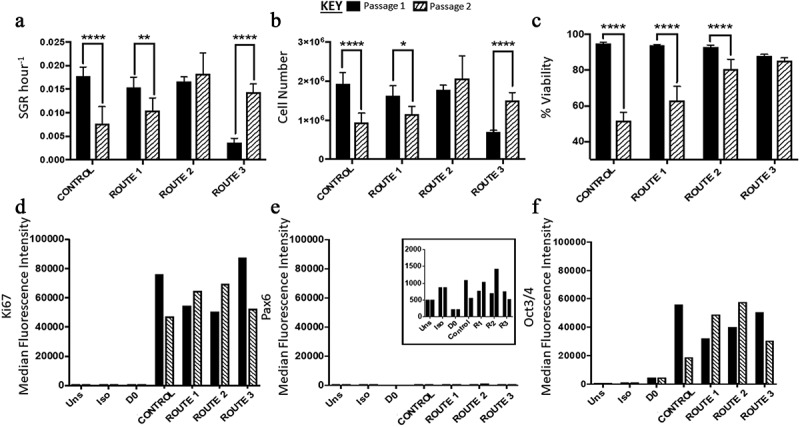
Analysis of H9 human embryonic stem cells (hESCs) growth dynamics for cells cultured under four different feeding regime routes. (a) Specific growth rate (SGR) decreased from passage 1 to 2 for the control and route 1 conditions, no significant difference was observed for route 2, while route 3 significantly increased from passage 1 to 2. (b) The same trend observed in the SGR was observed with the cell number yields from passage 1 to 2, i.e., only route 2 did not have a significant change in cell number between the two passages. (c) The control, route 1 and route 2 all had a decrease in cell viability from passage 1 to passage 2, which was not observed for route 3. Ki67 (d) and Oct2/4 (f) were both highly expressed in all the conditions. Pax6 median fluorescence intensity (MedFI) expression levels were very low in comparison to Oct3/4 and Ki67, inset shows the very low levels of Pax6 (**3E**), all conditions were under 1,500 for their MedFI values. Error bars indicate standard deviation, *n = *3. ‘*’ indicates p < 0.05, ‘**’ indicates p < 0.01, ‘***’ indicates p < 0.001, and ‘****’ indicates p < 0.0001. Uns = unstained; Iso = isotype; D0 = cells at day 0; R1 = route 1; R2 = route 2; R3 = route 3

Overall, there was a significant difference in the SGR of the different routes except between the control and route 1. Between passages each route differed in SGR from passage 1 to passage 2 apart from route 2 (Figure 3a). At passage 1 the significant differences were only between route 3 and the other conditions (route 3 having a lower SGR), however this changed at passage 2, with differences being between the control and route 2. Similarly, there was no significant difference in cell number for route 2 between passage 1 and 2. The control and route 1 both had a significant decrease in overall cell number, while route 3 had a significant increase (Figure 3b). Flow analysis revealed that both Ki67 and Oct3/4 were highly expressed in all conditions, while there was no expression of Pax6 (Figure 3e). Conversely, route 1 and route 2 both had increased Ki67 and Oct3/4 from passage 1 to 2 (Figure 3d and f). Cell viability in all routes of passage 1 was reported to be much higher in comparison to passage 2; however, route 3 exhibited similar viability for H9 cells in both passages (Figure 3c).

**Figure 4.**
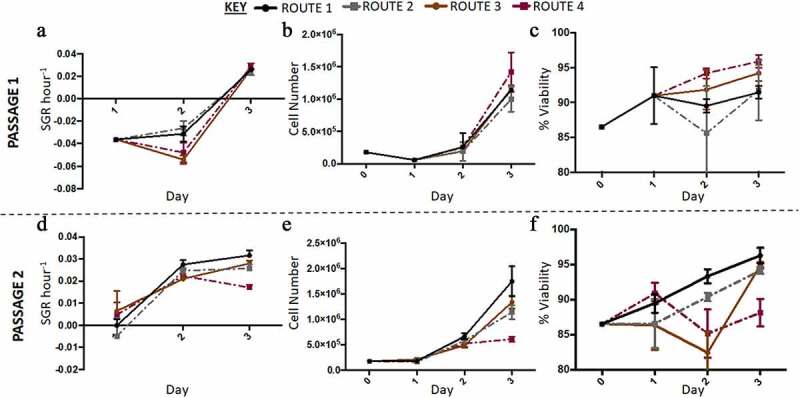
Growth dynamics of H9 cells with higher resolution cell count was carried out in the second run in Experiment B: the effect of feeding regimes. (a) Overall, the specific growth rate (SGR) was lower at passage 1, with all four conditions having negative SGRs until day three of passage 1. (d) At passage 2, route 2 and route 3 had a positive SGR from day one onwards, only route 3 had a significant decrease in SGR by day three of passage 2. At both passages the cell number increased from day 0 to day three, however at passage 1 (b) there was a decrease in cell number from day 0 to 1, not observed at passage 2 (e). Generally, cell viability increased throughout passage 1, except for route 1 which had a decrease on day two (c). At passage 2, route 2 and route 3 had variable cell viabilities from day to day, while the control and route 1 had increased cell viabilities from day to day (f). Error bars indicate standard deviation, *n = 3.*

**Figure 5.**
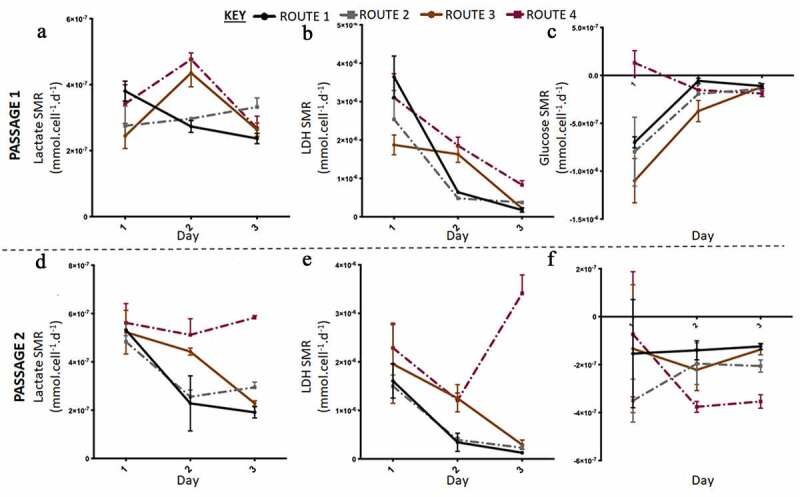
Lactate and lactate dehydrogenase (LDH) specific metabolic rate (SMR) data with higher resolution metabolite sampling was carried out in the second run in Experiment B: the effect of feeding regimes. Lactate SMR increased from passage 1 (a) to passage 2 (c), at passage 2 lactate SMR decreased for all routes except route 3. LDH SMR was highest for route 3 at passage 1 (b) and passage 2 (e), generally LDH SMR decreased for all routes except route 3 at passage 2. Glucose SMR of consumption was highest at day one passage 1 for all conditions, except route 3, passage 1 (**C**) and passage 2 (f). Error bars indicate standard deviation, *n = 3.*

In the second run of the experiment at a higher resolution, out of thaw the cells had low SGR and cell numbers (Figure 4a). At passage 1 the control and route 1 exhibited similar behaviors to each other and whilst route 2 and route 3 grouped together; thus, the difference in SGR was between the respective set of conditions. Route 3 had the highest cell number at the end of passage 1 (Figure 4b). Passage 2 demonstrated that route 3 was significantly different compared to the other conditions, as it had both the lowest SGR and cell number. The viabilities were reasonably similar at passage 1 across all the conditions investigated as no significant differences were obtained; however, at passage 2 the conditions had significantly different cell viabilities day to day and overall (Figure 4f). Both route 2 and route 3 dropped in SGR from day one to two and then increased from day to three at passage 1. There was no significant difference in expression levels between the routes and the passages for all three markers. Pax6 was not expressed in any of the different routes, whilst all the conditions highly expressed Oct3/4 and Ki-67 (Figure 3d,e and f). The rate of lactate production increased from day one to two and then decreased from day two to three in passage 1 for route 2 and route 3 cultures (Figure 5a). The rate of LDH production was at its highest in route 3 on day three at passage 2 (Figure 5d). There was no significant difference in the SMR for both LDH and lactate across the routes on day one at passage 2. However, on day three passage 2, route 3 was significantly higher than the other conditions for all three metabolites. The glucose SMR data demonstrates route 3 having a different metabolic profile in comparison to the other conditions (Figure 5c). At passage 1 day 1, glucose SMR for route 3 was positive, which suggests that SMR rate might be stimulated by the feeding regime in route 3 (Figure 5c). This was not observed in any of the other conditions. By day two at passage 2 the glucose SMR for route 3 aligned with the other conditions, into a consumptive state. All other routes initially began with a higher glucose SMR that sharply decreased by day two at passage 1. Meanwhile, the other conditions had a moderately undeviating glucose SMR throughout the passage, this is particularly the case for the control route.

## Experiment C: control of nutrient availability to reduce metabolic variation

**Figure 6.**
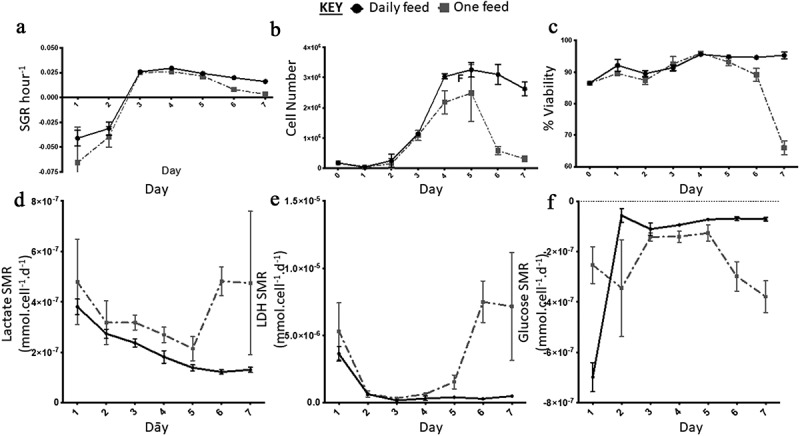
Growth dynamics. (a) Specific growth rate (SGR) increased from negative SGR to positive SGR from day three onwards peaking at day four for the daily feed (DF) condition and day three for the one feed (OF) condition. OF had a greater decrease in SGR over the seven-day culture period, SGR differed between the two conditions on day one (p = 0.0012). (b) Cell number decreased from day five onwards for both conditions, similar to the SGR OF has the most notable decrease in cell number and cell viability (c). From day four onwards there was a significant difference in cell number yield between the two conditions: day 4 (p < 0.0001); day 5 (p = 0.0002); day 6 (p < 0.0001); day seven (p < 0.0001). Error bars indicate standard deviation, *n = *6. Lactate SMR decreased throughout the passage for daily feed (DF), one feed (OF) decreased until day five to six when it increased significantly (p = 0.0079) (d). Lactose dehydrogenase (LDH) specific metabolite rate (SMR) was highest from day five to six for the OF condition, the DF condition had low levels of LDH production over the seven-day culture period (e). Significant differences in SMR between the two conditions for both lactate and LDH were observed at day six (p < 0.0001) and day seven (p < 0.0001). Glucose SMR was high at day one for the DF condition after which it significantly decreased from day two onwards and plateaued (f). Error bars indicate standard deviation, *n = *3

**Figure 7.**
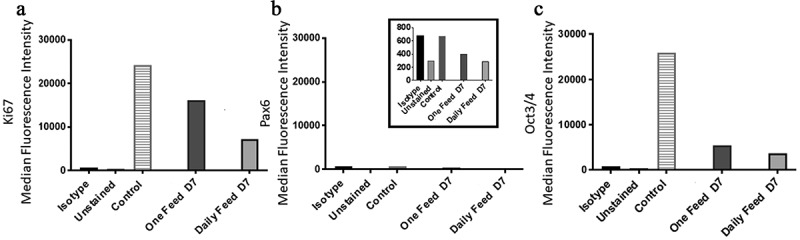
Ki67 and PAX6 and OCT3/4 median fluorescence intensity (MedFI) values. Ki67(a) and OCT3/4 (c) were both highly expressed in both conditions. (b) PAX6 expression levels were lower in comparison to OCT3/4 and Ki67, inset shows the very low levels of PAX6, all conditions were under 800 for their MedFI values. Ki67 and OCT3/4 decreased from day six to seven, resulting in lower expression level overall when compared to the control (day 0) and both conditions at day seven

On day one and two both the DF and OF conditions had a negative SGR which increased significantly from day two to three (Figure 6a). The two conditions varied in terms of cell number from day three onwards, although the trend remained comparable; the DF condition started to plateau at day 4, as there was no significant difference in SGR and cell yield from day four onwards (Figure 6a and b). Meanwhile, the OF condition significantly dropped in cell number from day five, which was also accompanied by a decline in cell viability down to 65%±2.1 by day seven (Figure 6c). The metabolite analysis revealed that the rate of lactate and LDH production decreased over the seven days for the DF condition (Figure 6d and e). LDH levels decreased more dramatically between day one and two and reached a plateau from day three onward while, lactate levels continued a downward trend over the seven-day experiment (Figure 6d and e). A similar trend was observed for the OF condition until day 5 where the rates of production significantly increased from day five to six and then stabilized from day six to seven (Figure 6e). Glucose SMR was high at day one for the DF condition after which it significantly decreased from day two onwards and plateaued (Figure 6f). The OF condition demonstrated a different trend, with greater fluctuation; the glucose SMR was initially low (day one), which increased (day two to three) prior to plateauing at a lower rate (day three to five) compared to the higher rate at day five to seven (Figure 6f). The flow marker analysis of MedFI revealed that Ki67 and Oct3/4 expression decreased overall between day 0 (the control, Figure 7) to day seven and from day six to seven in both conditions; the biggest decrease in MedFI was between day 6 to 7 of the DF condition for Oct3/4 (Figure 7). There was a very low expression of Pax6 in all conditions across the sampling points (Figure 7b).

## Discussion

Understanding the culture dynamics of pluripotent cells is important to inform cell culture procedures, since input cell material such as pluripotent cells can have an impact on the output cells obtained through the manipulation and differentiation of the former [37,38]. For example, cell culture parameters are often non-standardized and lack defined protocols which results in process variation. Consequently, process tolerances and specific quantifiable quality metrics are difficult to prescribe as the outputs of these non-standardized processes are variable. This ultimately causes inconsistency in outputs such as cell growth rates, cell metabolism and cell phenotype [39]. Typically, published cell culture and differentiation protocols provide vague statements regarding the quality of input cells, with one protocol stating that the cell differentiation process should start with ‘good-quality starting material’[28]. However, there is currently no quantifiable data or standardized assays related to what ‘good quality’ explicitly refers to. At most, published protocols provide a vague description as to how the cells should appear using visual criteria [28]. A substantial reliance upon cell ‘visuals’ is inherently flawed in that they are open to operator interpretation, experience and training; thus, they are highly subjective [8]. It is worth noting that, while the use of visual inspection cannot be eradicated and is in fact useful, it should be cross validated with quantitative methods that are standardized, thus facilitating reproducibility and comparability.

Furthermore, critical process parameters (CPPs) including medium exchange time points, observed confluency and passage time points are subject to operator interpretation. This issue is prevalent in many published protocols [15,39–44]; for instance, a range of passage timings, i.e., three to five, is suggested for cells to reach confluency. Additionally, defined seeding densities are absent in many protocols, with the use of split ratios being commonplace [9,43,45–48]. The issues are problematic for the standardization of any cell manufacturing protocol since the lack of defined parameters and procedures ultimately results in product variation. The lack of standardization can result in small changes to culture conditions that have a significant effect on cell characteristics such as growth, viability, and phenotype. Therefore, it is vital that process conditions are standardized and controlled to obtain output cells that are within a desired CQA specification range. If the variation caused by non-standardized culture condition is out of the specification range, it can nullify batches, hinder product release and waste financial and labor resources [16,17].

CQAs are functionality-based characteristics that are fundamental to the clinical response. Characterization studies provide information regarding what ‘good quality’ starting materials should be for the differentiation process. This includes the use of metrics including minimal cell viability, SGR, and levels of phenotypic markers (including Ki67 and OCT3/4), required to quantify a pluripotent expansion culture. The addition of these metrics allows for quantifiable benchmarks to permit standardized input of cell material for differentiation in vmDA neuroprogenitors. For CTPs such as vmDA cells for transplantation it is crucial that the cells are well characterized as subtle differences appear between DA neurons and neighboring cells. The work presented here was concerned with the process development of the most recent Lund University differentiation protocol [28]. The protocol is designed to produce vmDA neuroprogenitors which have been previously shown to innervate into the striatum [30]. A better understanding of cell functionality may predict long-term graft outcomes [49]. This will ultimately accelerate the progression of CTPs toward clinical translation. Furthermore, a greater understanding and predictability of cell maturation and functional properties will facilitate the use of autologous or individually matched cells for transplantation [49]. Currently, further investigation is required to enable a full development process following a risk-based approach.

Some of the aforementioned sources of variation can be controlled, minimized and/or measured *via* the implementation of robust and standardized protocol parameters. The work presented here demonstrates the importance of defined seeding densities, feeding regimes and process understanding, including recovery from cryopreservation, to facilitate more efficient and standardized cell culture protocols that reduce process variation. The increase in seeding density and the inclusion of a recovery passage following cryopreservation resulted in improved proliferation rates, cell number and viability. The higher resolution growth curve studies (run 2) which included the experimental conditions in triplicate and duplicate counting samples demonstrated that one day after seeding the cells were typically at a low or negative growth rate which increases significantly from day 2 onwards which is observed in other growth curve studies of hESCs and human mesenchymal stem cells [50–52].

The advantage of streamlining and optimizing protocols is that it increases process efficiency whilst producing a translatable, robust protocol for clinical manufacturing. This is achieved by reducing potential sources of human-based errors such as a reliance on visual criteria, a reduction in uncontrolled parameters including reagent batch-to-batch variation, and increased efficiency. The revised protocol in this work used GMP reagents to adapt the Lund protocol for clinical manufacturing. This also supports the reduction of inter-lab variation whilst facilitating process transfer, permitting expansion and upscale and multiple sites. Increased protocol efficiency and scaling up to larger culture vessels is economically favorable and manageable in terms of scale and manipulation.

## The influence of seeding density during pluripotent cell expansion

Cells seeded at 10,000 cells/cm^2^ did not perform as well as those seeded at the higher densities, the 10,000 cells/cm^2^ conditions had lower cell viabilities and took longer to reach confluency as their SGR was lower in comparison. This illustrates that cell seeding density influences the growth rate of cells during pluripotent expansion; therefore, it is a CPP that should be controlled to ensure process standardization and reproducibility [53,54]. Controlling a parameter such as seeding density can aid in the reduction of process variation, which is necessary for successful translation of CTPs [16,55,56]. The cells performed better after at least one passage, in terms of cell number and a shorter duration to reach confluency (three days instead of four days). Thus, a ‘pre-passage’ should be utilized prior to further experiments and differentiation; allowing the input cell material to stabilize and therefore result in less variation. The effects of cryopreservation and the putative ‘lag’ following cryopreservation are discussed in the literature [57,58], which supports the necessity of culture processes that allow the cells to acclimatize and stabilize in terms of growth rate, i.e., a recovery passage. This information is integral to designing manufacturing processes and protocols, for instance the additional passage before the cells are further utilized has an impact on labor, time and reagent resources all of which ultimately influence the cost of the process [59,60].

## Streamlining of feeding regimes

Analysis of the different feeding regimes demonstrated that route 2, whereby the cells were only subjected to a medium exchange on day two, resulted in cells with higher or comparable cell viability, SGR and cell number to the control condition which was subjected to a daily medium exchange. Route 2 also exhibited an increase in Oct3/4 and Ki67 expression between the two experimental passages. In general, the control and route 1 behaved similarly, while route 2 and route 3 were comparable to each other; this could be attributed to the time points in which the ROCK*i* was removed. ROCK*i* reduces dissociation-induced apoptosis in hESC cultures and is a putative cell survival and proliferation inducer [61–64]. The control and route 1 both had ROCK*i* removed on day one when they were subjected to a medium exchange. In the second run of the experiment, it was evident that the route 3 was significantly different to the other conditions in terms of SGR, cell number yield and its metabolite profile. This was typically in an undesired manner as evidenced by a lower SGR, cell viability and higher LDH SMR. At passage 1 the glucose SMR was similar amongst the culture conditions apart from route 3 at day 1. The glucose SMR was higher at day 1 which can be attributed to the energy requirements of the cells while attaching and initiating the proliferation stage, during passage 1 this trend was similar for all the conditions, with the exception of route 3. These results were in line with published studies that showed that accumulation of excreted lactate decreases the growth rate and provision of glucose and that eliminating lactates are important factors in the expansion of induced pluripotent stem cells (iPSCs) when reduced culture medium is used [65]. Furthermore, the results of lactate reduction were comparable with Hong et al’s., findings that demonstrated a lactate shift with less lesser lactate production in the exponential growth phase in Chinese hamster ovary (CHO) cell cultures [66]. Thus, this emphasizes the importance of the provision of a balanced media composition which enables cells to favor the particular metabolic pathways for the desired lactate shift [66].

However, by passage 2 there was less uniformity, proposedly due to the previous culture condition imposed at passage 1. The control route and route 2 showed little fluctuation in glucose SMR at passage two, while route 1 and route 3 were more variable. This illustrated the difference that one passage can have from another in terms of cell metabolism, thus highlighting the needs to better understand cell culture dynamics to produce protocols that adequately consider the behavior of the cells.

These results suggest that the cells do not have to be subjected to a medium exchange daily, as route 2 with only one medium exchange resulted in cells with the desired characteristics when compared to the control of daily medium exchange. However, the cells do need to be subjected to a medium exchange to avoid the decrease in SGR and cell viability and higher LDH SMR observed in the route 3 conditions. Similar to experiment A, during passage 1 at day two the cells were still at a negative growth rate, while at passage 2 on day two they were at their peak. This further highlights the necessity of a recovery passage. It is necessary to note that the present work utilized a three-day protocol; thus, further analysis and optimization of medium exchange frequency is required for a longer passage length.

## Control of nutrient availability to reduce metabolic variation

Experiment C aimed to identify the density and nutrient limitation points for the cells to establish an optimal cell culture protocol for the H9 hESCs. The length of an optimal culture period for cells seeded at 20,000 cells/cm^2^ straight out of cryopreservation was shown to be four days. For cells seeded at 20,000 cells/cm^2^ and cultured immediately following resuscitation, growth limitation based on both density and nutrient availability was observed. However, the total cell numbers suggest that cell growth starts to decline from day five onwards, with nutrient availability factoring in from day three onwards as the DF (density inhibition) and OF (nutrient availability) conditions varied at this point. This was due to the decrease in SGR and cell viability from day four onwards, caused by the high levels of confluency resulting in cell death, inferably due to competition for nutrient availability.

The rate of metabolite production shows the impact of nutrient availability on cell growth, i.e., in general, the glucose SMR per cell decreases as less nutrients were available during the exponential growth phase due to more cells proliferating [67–69]. In the OF condition, the impact of restricted nutrient availability as the cells reach growth limitation was highlighted, at day 5 the significant decrease in SGR, cell number and decline in cell viability was accompanied by an increase in the rate of production of both lactate and LDH. The increase in LDH production shows that the cells were starting to enter the inhibition stage prior to cell death and enter the decline phase [65,68]. The increase in lactate production can be attributed to having less cells in the same medium from day one (OF condition) leading to an accumulation of lactate since no medium exchange was performed. Therefore, the per cell rate of lactate and LDH production significantly increases at the point of the highest decline in cell number (day five), despite there being less cells and less nutrient metabolism. By day seven, high levels of metabolic activity variation were observed in the OF conditions, this was ascribed to the high levels of cell death at this time point. Day two was shown to have the highest levels of lactate production, as such, a medium exchange to remove the accumulated lactate would be beneficial to the cells [65,66,68,70]. Day 2, 3 and 4 which made up the exponential growth phase show low SGR, cell viability and cell metabolic variation illustrating that the cells were highly synchronous; thus, day 4 was the optimal time point to harvest the cells for further use. In contrast, from day five onwards the cells were highly variable in terms of their metabolite rate and the cells exhibit decreased cell viability.

Comparably, a study carried out by Singh et al. reported a correlation between differentiation and an earlier plateauing of exhaustive glycolysis, decreased lactate production, lower metabolite consumption and reduced cellular proliferation in ESCs [68]. Their findings further indicated that lower initial cell density led to an increase in the rate of glycolysis, metabolite utilization including glucose consumption and lactate secretion, and proliferation over a similar culture period [68]. This illustrates the need to ensure that cells do not enter such a detrimental phase, as the resultant cells would be highly variable and inadequate for further use, especially as an input material for a potentially therapeutic product. The plateauing of the glucose SMR at a lower rate in the DF can be attributed to the cells entering a phase of stable growth and proliferation, due to the daily addition of glucose. Therefore, the rate of consumption was lower compared to the OF condition where the amount of glucose available was lower (due to no medium exchange) thus, the observed rate was higher for a similar given number of cells. The fluctuation in glucose SMR for the OF condition illustrates the need to feed the cells in order to reduce metabolic variation. The difference in glucose concentration between the two conditions could explain the observed differences in glucose SMR. For the OF condition, the limited availability of glucose could result in higher glucose SMR due to a competing and stress effect from the cells. In contrast, the excess glucose concentration in the OF condition, particularly for as the SGR plateaus could result in a lower glucose SMR due to glucose intolerance [71]. These findings are in agreement with the results of Du et al. that showed the initiation of oxidative stress-induced glycolysis due to the reduced glucose supply (less frequent feeding) in hESC culture [71].

This information highlights the levels of process understanding developers require in order to produce well-informed protocols that are efficient, reproducible, address the needs of the cells whilst retaining the CQAs without under or over feeding the cells [65,72]. This is integral for processes that use the cells as a therapeutic product, such is the case for cell therapy products including Yescarta and Kymriah [73–75]. Other CPPs that should be addressed in the standardization of cell culture protocols include: feeding frequency for long passage protocols; the percentage of medium exchanges; pH ranges; duration of ROCK*i* supplementation (for protocols that use ROCK*i*); cell passage number; and the experimental design employed [11,16,17,55,62].

## Optimized culture parameters for culture of H9 hESCs

The results determined that a set of parameters: a seeding density of 20,000 cells/cm^2^, a four-day culture period, or three-day culture period following a recovery passage (out of cryopreservation) was optimal for H9 hESCs to reach confluency, while maintaining high cell viability and the desired pluripotent phenotypic markers. These parameters were identified through all three experiments, the growth limitation experiment (Experiment C) highlights the negative SGR at day one and two for cells in their first passage from cryopreservation. It was only from day three onwards that the SGR started to increase, suggesting that the cells should have at least one passage prior to experimentation to allow their SGR to stabilize.

Establishing that the cells need a recovery passage represents a key element for manufacturing considerations; as resources will need to be adjusted, while taking into account the impact of the recovery passage on overall production timelines and product costs [76–78].

Future work should investigate the effects CPP and culture dynamics on other cell characteristics such as gene expression. Furthermore, validation of CPPs will need to be performed in order to obtain process tolerances that ensure achievement of the desired cell outputs (CQAs). Understanding cell dynamics such as the requirement of a ‘prior passage’ can facilitate developers in ensuring that their processes are not subject to failure due to the use of cells that are not in their optimal state. This knowledge can safeguard manufacturing processes and avoid loss of productivity and sunk costs due to process batch failures [17].

hESC lines including H9s are considered to be very similar in terms of the expression of pluripotency markers and their ability to differentiate. However, evidently variation between lines including growth rate, genetic and epigenetic stability in long-term cultures also exist [79–81]. Notably, there is a need for much more comprehensive, characterization of hESC lines and protocol standardization than is currently being undertaken to enable the developers to assess the true potential of hESCs for therapeutic applications. Nevertheless, the implementation of a wide range of culture systems and protocols including the use of feeder cells, culture media, feeding regime and passage methods to derive lines is still inadequate in interpretating inter-line differences and to distinguish between the true impact of inherent genetic variation and environmental ‘programming’ of the cells [19]. To date, there are still no established standard conditions for H9 expansion that are optimal or generically applicable across lines [80]. This is due to the fact that the characteristics described for cells that were cultured in certain conditions (and at a specific passage number), therefore, can only be applicable to those specific cells assayed within an individual laboratory. Despite some success in standardizing conditions between independently derived lines for maintenance and cardiomyocyte differentiation, the culture systems are often not transferable or have proven difficult to transfer across different laboratories [42,82].

Nevertheless, this information can only be obtained by rigorous experiments that analyze the effect of parameters such as density and feeding regime on cell characteristics and behaviors such as metabolic rates and phenotype expression. Being equipped with such information will allow developers to better translate their protocols into manufacturing process that are well informed, standardized, and robust enough to control/reduce process variation. This should be done early on during product and process development in order to (1) accrue high-resolution data as early as possible; (2) preempt process challenges and resolve them prior to clinical and manufacturing translation and (3) ensure that the former two points have been addressed to ensure regulatory requirements are met, thus facilitating regulatory approval [12,16,17,55,83]

## Conclusion

Adequate understanding of culture dynamics permits developers to optimize protocols and feeding regimes to suit-specific cell requirements. This work provides key culture conditions crucial in optimized and cost-effective bio processes in comparison to generic protocols and ad hoc feeding regimes that are typically used [53,84]. A range of targeted experiments were used to ascertain optimal protocol parameter(s) for the pluripotent expansion of H9 hESCs. Results revealed that optimizing key parameters including feeding regime and cell density leads to an improved SGR, high cell viabilities and elevated expression of pluripotent and proliferation markers. Our findings highlight the significance of cell dynamics knowledge to aid the development and application of streamlined protocols in highly optimized, robust, and reproducible cell manufacturing processes [53,85].

## Research highlights

- Pluripotent culture require standardization to ensure consistent input prior to differentiation
- Understanding the cells culture dynamics can be used as a tool in protocol improvement
- Defining critical process parameters including feeding regime is crucial in process optimization
- Cell dynamics knowledge can aid more robust and reproducible cell manufacturing processes
